# Supplementary material for: CD163-Expressing Porcine Macrophages Support NADC30-like and NADC34-like PRRSV Infections
Source: Viruses. 2022 Sep 16;14(9):2056. doi: 10.3390/v14092056 (PMC9505768; doi:10.3390/v14092056)
Supplement: Supplementary file 1 [file viruses-14-02056-s001.zip › viruses-1897009-Supplementary Materials.pdf]

**Table S1.** PCR primers used for the construction of PRRSV JSTZ1712-12 infectious clones.

| Primer Names | Primer Sequences * |
|---|---|
| JSTZ1712-12-*Pac*I-F1 | AGCTCGTTAATTAATACATGACGTATAGGTGTTGGCT |
| JSTZ1712-12 -*Afl*II-R1 | CATAGGTGCTTAAGTTCATTACCACCTGTAACGGAT |
| JSTZ1712-12 -*Afl*II-F2 | ATCCGTTACAGGTGGTAATGAACTTAAGCACCTATG |
| JSTZ1712-12 -*Asc*I-R2 | CCTTTCTGGCGCGCCCGAAAC |
| JSTZ1712-12 -*Asc*I-F3 | GTTTCGGGCGCGCCAGAAAGG |
| JSTZ1712-12 -1R3 | ***AGCGAGGAGGCTGGGACCAT****GCCGGC*TTTTTTTTT TTTTTTTTTTTTAATTACGGCCGCATGGTTCT |
| JSTZ1712-12 -*Not*I-2R3 | ACAGGCGGCCGC*GTCCCATTCGCCATTACCGAGGGG ACGGTCCCCTCGGAATGTTGCCCAGCCGGCGCC****AGC GAGGAGGCTGGGACCAT***# |
| dsRed- *Kpn*I-F | ATTGAAGGTACCGCCACCATGGCCTCCTCCGAGGA |
| dsRed- *Bcl*I-R | TGCCGCGGAATGATCACTACAGGAACAGGTGGTGGC |

* The restriction enzyme sites used for cloning purposes were underlined. # The hepatitis D virus ribozyme sequence was shown in italic and the overlapped region was highlighted in bold.

**Table S2.** Primers and probes of qPCR used in this study.

| Primers/Probes | Sequences (5′–3′) | Genes |
|---|---|---|
| | | ORF7 |
| PRRSV ORF7-F | ATAACAACGGCAAGCAGCAG | ORF6 |
| PRRSV ORF7-R | CTCTGGACTGGTTTTGTTG | |
| PRRSV2-UF | TTGTGCTTGCTAGGCCGC | |
| PRRSV2-UR | ACGACAAATGCGTGGTTATCA | |
| PRRSV-UProbe | FAM-TCTGGCCCCTGCCCA-MGB | |

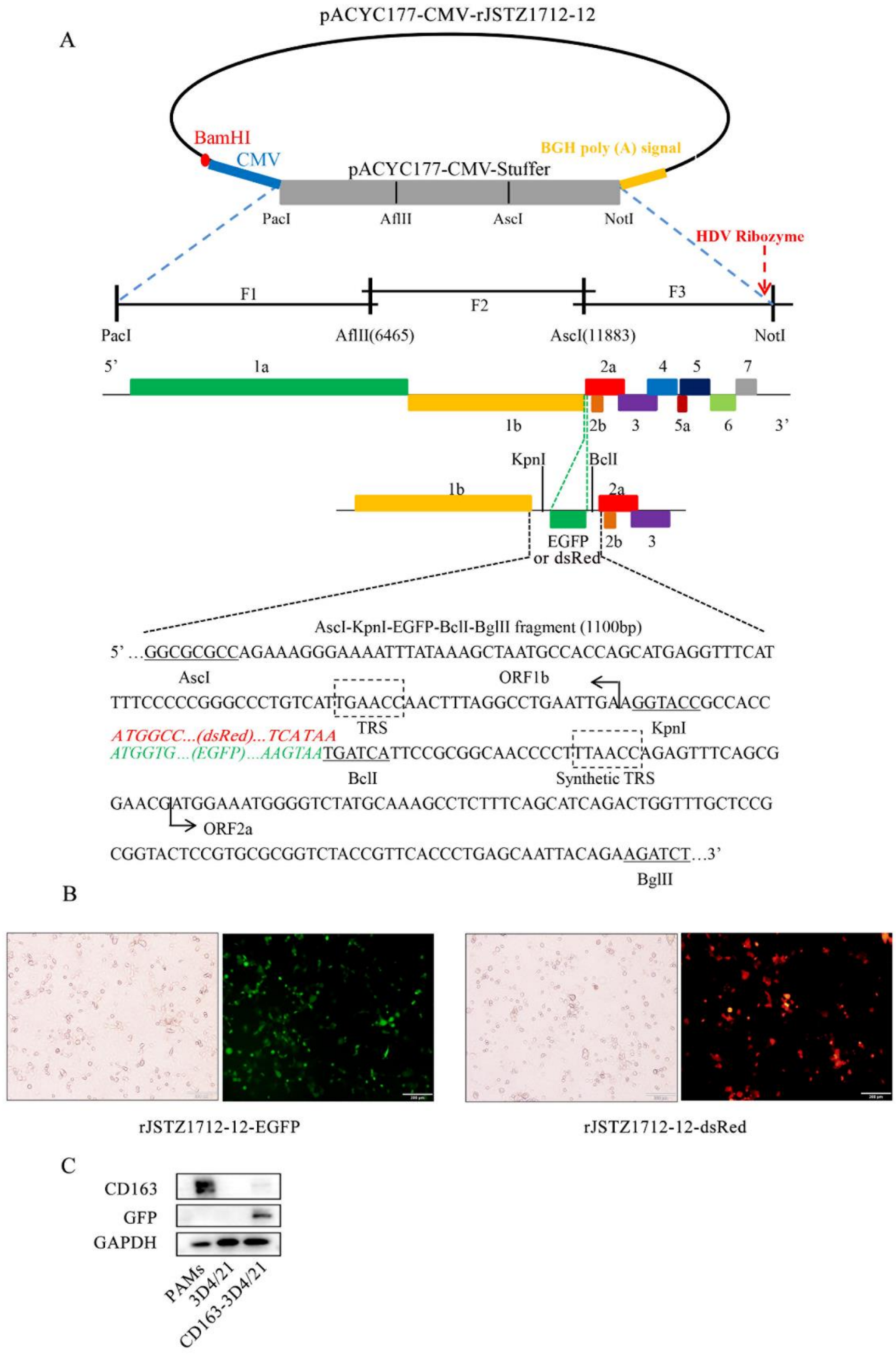


**Figure S1.** (**A**) Strategies to construct the full-length cDNA clones of HP-PRRS JSTZ1712-12 strains carrying EGFP or dsRed. The strategy was as we described previously (*Transbound Emerg Dis*. 2020; 67:1820–1827. *Vaccines* (Basel). 2021; 9(10):1176. *Viruses*. 2021; 13(9):1829). The pACYC177-CMV-Stuffer fragment including the unique restriction enzymes was shown in the top part. The three overlapped fragments of the full JSTZ1712-12 genome were produced by PCR amplification and shown in the middle part. The *Asc*I-*Kpn*I-EGFP (or dsRed)-*Bcl*I-*Bgl*II fragment and the strategy to insert EGFP or dsRed gene into the rJSTZ1712-12 clone were shown in the bottom part. (**B**) Characterization of the rescued rJSTZ1712-12-EGFP and rJSTZ1712-12-dsRed PRRSV. Marc-145 cells were infected with PRRSV-EGFP/dsRed at an MOI of 0.1, respectively. CPE were observed and the GFP/dsRed signal was visible under fluorescence microscope at 48 hpi. (**C**) CD163 expressions in different porcine macrophages. Primary porcine alveolar macrophages (PAMs), 3D4/21 and CD163-3D4/21 cells were detected for CD163 expressions by Western blotting using CD163 rabbit pAb. The ectopic CD163 expression was also monitored by GFP mouse mAb.
